# The role of lipid metabolism in neuronal senescence

**DOI:** 10.1002/2211-5463.70181

**Published:** 2025-12-12

**Authors:** Dikaia Tsagkari, Eleftheria Panagiotidou, Nektarios Tavernarakis

**Affiliations:** ^1^ Department of Basic Sciences School of Medicine, University of Crete Greece; ^2^ Institute of Molecular Biology and Biotechnology, Foundation for Research and Technology‐Hellas Greece; ^3^ Department of Biology School of Sciences and Engineering, University of Crete Greece

**Keywords:** ageing, autophagy, lipid metabolism, mitochondria, neurodegeneration, neuronal cells, senescence

## Abstract

Senescence is a complex cellular state characterised by irreversible growth arrest and metabolic reprogramming. In neurons, senescence has been mainly observed in the context of ageing and age‐related neurodegeneration. Lipid metabolism plays a critical role in cellular homeostasis, with emerging evidence suggesting that alterations in lipid species, including fatty acids, cholesterol, sphingolipids and phospholipids, fundamentally drive or contribute to the senescent phenotype in both neuronal and non‐neuronal cells in the brain. Namely, changes in lipid species levels result in the accumulation of lipid droplets (LDs), leading to dysregulation of membrane dynamics, and in turn to the production of bioactive lipid mediators, which collectively shape the senescence‐associated secretory phenotype (SASP) in the brain. In this review, we describe the cell type‐specific patterns of lipid dysregulation in neurons, astrocytes, microglia and other glial cells during senescence, highlighting the role of key lipid species and their association with senescence markers and phenotypes. Furthermore, we discuss the bidirectional relationship between lipid metabolism and mitochondrial dysfunction in cellular senescence. We also examine the molecular mechanisms through which lipid metabolic pathways can orchestrate neural senescence and their contribution to ageing and age‐related neurodegenerative disorders, such as Alzheimer's disease and Parkinson's disease. Finally, we review emerging therapeutic strategies targeting lipid metabolic pathways to modulate neural senescence and potentially ameliorate age‐associated brain pathology.

## Graphical abstract

Disrupted lipid metabolism, through alterations in lipid species or lipid droplet accumulation, can drive neuronal senescence. However, lipid dyshomeostasis can also occur alongside neuronal senescence, further amplifying tissue damage. Delineating how lipid‐induced senescence emerges in neurons and glial cells, and how it contributes to ageing and neurodegenerative diseases, is essential for developing effective therapeutic strategies.

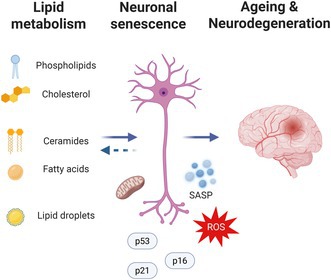

**Abbreviations**
ABCA1: ATP‐binding cassette transporter A1
ABCG1: ATP‐binding cassette transporter G1
ACSL1: acyl‐CoA long‐chain family member 1
AD: Alzheimer's disease
AEP: asparagine endopeptidase
ALDH: aldehyde dehydrogenase
AMD: age‐related macular degeneration
AP1: activating protein 1
APOE: apolipoprotein E
ATM: ataxia‐telangiectasia mutated
ATP: adenosine triphosphate
ATR: ATM‐ and Rad3‐related
BDNF: brain‐derived neurotrophic factor
BMDM: bone‐marrow‐derived macrophage
BNIP3: BCL2/adenovirus E1B 19 kDa protein‐interacting protein 3
Cav1: Caveolin‐1
CDK: cyclin‐dependent kinase
CerS1: ceramide synthase 1
CHK1/2: checkpoint kinases 1 and 2
CNS: central nervous system
DAG: diacylglycerol
DGAT2: diacylglycerol O‐acyltransferase 2
FA: fatty acid
FAO: fatty acid oxidation
G3P: glycerol‐3‐phosphate
GBA: glucosylceramidase beta 1
GCase: glucocerebrosidase
GluCer: glucosylceramide
GluSph: glucocylsphingosine
GPNMB: glycoprotein nonmetastatic melanoma protein B
GSH: glutathione
HMGCR: 3‐hydroxy‐3‐methylglutaryl (HMG)‐coenzyme A (CoA) reductase
iNSC: induced neural stem cell
ISR: integrated stress response
LD: lipid droplet
LDL: low‐density lipoprotein
LDL‐C: low‐density lipoprotein cholesterol
LSR: lipolysis‐stimulated receptor
LXR: liver X receptor
MMP: matrix metalloproteinase
mTOR: mammalian target of rapamycin
mTORC1: mammalian target of rapamycin complex 1
MUFA: monounsaturated fatty acid
NRSF/REST: neuron‐restrictive silencer factor
nSMase2: neutral sphingomyelinase 2
PC: phosphatidylcholine
PD: Parkinson's disease
PE: phosphatidylethanolamine
pEtN: phosphoethanolamine
PMS: progressive multiple sclerosis
PNS: peripheral nervous system
PUFA: polyunsaturated fatty acid
Rb: retinoblastoma
ROS: reactive oxygen species
RXR: retinoid X receptor
SASP: senescence‐associated secretory phenotype
SATB1: Special AT‐rich sequence‐binding protein 1
SA‐β‐gal: senescence‐associated β‐galactosidase
SPT: serine palmitoyltransferase
TAG: triglyceride
TrkB: tropomyosin receptor kinase B
UDP: uridine 5′‐diphospho‐glucuronosyltransferase

Cellular senescence, initially defined in 1961 [1], represents a state of stable, irreversible cell cycle arrest that cells can enter in response to stressors, developmental cues or intrinsic timing mechanisms, such as telomere shortening [2, 3, 4, 5]. Depending on the context, senescent cells can promote inflammation and cellular dysfunction or tissue regeneration and repair [4, 6, 7]. Senescent cells are defined by four hallmark features: irreversible cell cycle arrest, persistent DNA damage, a senescence‐associated secretory phenotype (SASP) and altered metabolism [2]. The SASP comprises a diverse array of bioactive molecules, including pro‐inflammatory cytokines and chemokines, immune modulators, angiogenic factors and matrix metalloproteinases (MMPs) [8]. Given the constant changes an organism faces throughout its lifetime, senescence is a cellular safety mechanism, with important roles in ageing, age‐related diseases, cancer and therapeutic strategies [9, 10, 11].

Intriguingly, it has been revealed that postmitotic cells, like neurons, can also undergo senescence [12]. This was unexpected, given that neurons typically reside in the G0 phase and proliferation arrest is an important feature of cellular senescence. However, studies have shown that neurons can re‐enter the cell cycle to repair DNA damage, and this process is accompanied by transcriptional changes characteristic of cellular senescence [13, 14, 15]. Neuronal senescence is primarily defined by changes in neuronal morphology, epigenetics, metabolic reprogramming and the release of SASP [16]. The emergence of senescence‐like phenotypes in neurons is indicated by increased expression of senescence biomarkers, such as the cyclin‐dependent kinase inhibitors p16^INK4a^ and p21^CIP1^ [12, 17, 18]. p21 is transcriptionally activated by p53, following DNA damage, and suppresses multiple cyclin‐dependent kinase (CDK) complexes, while p16 inhibits CDK4/6 to enforce retinoblastoma (Rb)‐dependent cell cycle arrest. The upregulation of p16^INK4a^ and p21^CIP1^ in neurons is predominantly linked to persistent DNA damage, which drives the stabilisation of senescence‐like states. The lysosomal enzyme senescence‐associated β‐galactosidase (SA‐β‐gal), an established senescence marker in other cell types, may be unreliable for neuronal senescence, as it accumulates in young mice without correlation to DNA damage [19], requiring evaluation alongside additional markers for accurate identification. To date, evidence suggests that neuronal senescence arises in response to or alongside a stress response [20].

Senescent cells are marked by profound alterations in lipid metabolism and dynamics, but the link between lipid metabolism and neuronal senescence remains poorly understood [2, 21]. Lipids comprise a wide range of species, including fatty acids, phospholipids, cholesterol and sphingolipids, that are essential for cell structure, function and trafficking [22, 23]. This is particularly relevant to the brain, which is second only to adipose tissue in lipid content and harbours a remarkably diverse lipid landscape; more than 700 distinct lipid species have been identified in the mouse brain alone [24, 25]. Among these, cholesterol metabolism plays a central role in shaping membrane composition, supporting myelin formation and regulating synaptic function and signalling [26, 27, 28, 29].

In this review, we systematically examine the emerging role of lipid metabolism in cellular senescence, integrating recent discoveries across neuronal and glial cell populations. We discuss the specific lipid species and metabolic pathways which regulate senescence induction or maintenance, explore the bidirectional relationship between lipid dysregulation and mitochondrial dysfunction, and evaluate the contribution of senescence‐associated lipid alterations to ageing and age‐related neurodegenerative disease pathogenesis. Finally, we consider the therapeutic potential of targeting lipid metabolism to modulate neuronal senescence and promote healthy brain ageing.

## The emerging role of lipid metabolism in cellular senescence

Alterations in lipid species have been consistently observed during senescence and are well documented in many recent and comprehensive reviews [21, 30, 31, 32, 33]. The accumulation of lipid droplets (LDs), dynamic cellular organelles that contain a neutral lipid core surrounded by a phospholipid monolayer, has also been documented in senescent cells [34, 35, 36, 37]. Furthermore, elevated expression of lipid metabolism‐associated enzymes and genes, such as aldehyde dehydrogenase (ALDH) in tumour cells [34] and *ASAH‐1* in fibroblasts [38], has been reported following the induction of senescence. Integrative transcriptomic and metabolomic analyses have revealed increased levels of glycerol‐3‐phosphate (G3P) and phosphoethanolamine (pEtN), which contribute to LD accumulation and altered phospholipid dynamics in senescent cells [36]. These findings align with the energy demands of senescence and the function of the SASP, which require extensive lipid and membrane remodelling [31]. Moreover, secretory phospholipase A2, which hydrolyses membrane phospholipids and acts upstream of eicosanoid production, induces cellular senescence in fibroblasts by increasing reactive oxygen species (ROS) levels [39]. The resulting oxidative stress activates a DNA damage response, which leads to activation of ataxia‐telangiectasia mutated (ATM) kinase and subsequent phosphorylation of p53 at serine 15, triggering senescence [39]. However, it remains unclear whether lipid dysregulation is indeed a driver or just one of the consequences of the senescent state.

Metabolic reprogramming is a hallmark of cellular senescence [2]. Senescent cells are characterised by dysfunctional mitochondria, dysregulated adenosine triphosphate (ATP) production and elevated levels of ROS, promoting lipid damage and further contributing to senescence progression [32]. Notably, DNA damage has been shown to induce mitochondrial alterations via BCL2/adenovirus E1B 19 kDa protein‐interacting protein 3 (BNIP3), leading to increased fatty acid oxidation (FAO), which drives cellular senescence [40]. In contrast, induction of senescence in hepatocytes involves mitochondrial dysfunction accompanied by a diminished capacity for FAO [35]. Thus, changes in lipid metabolism may not act as an independent driver of senescence but can exert significant influence through an interplay with mitochondrial function.

## Lipid signalling and senescence in the brain

To date, most studies investigating the link between lipid remodelling and cellular senescence have focused on fibroblasts and cancer cells. Recently, however, research has begun to extend to neurons. The brain exhibits a lipidome, that is conserved across species and, distinct from non‐neural tissues, with each region and cell type showing unique signatures [25, 41].

**Fig. 1:**
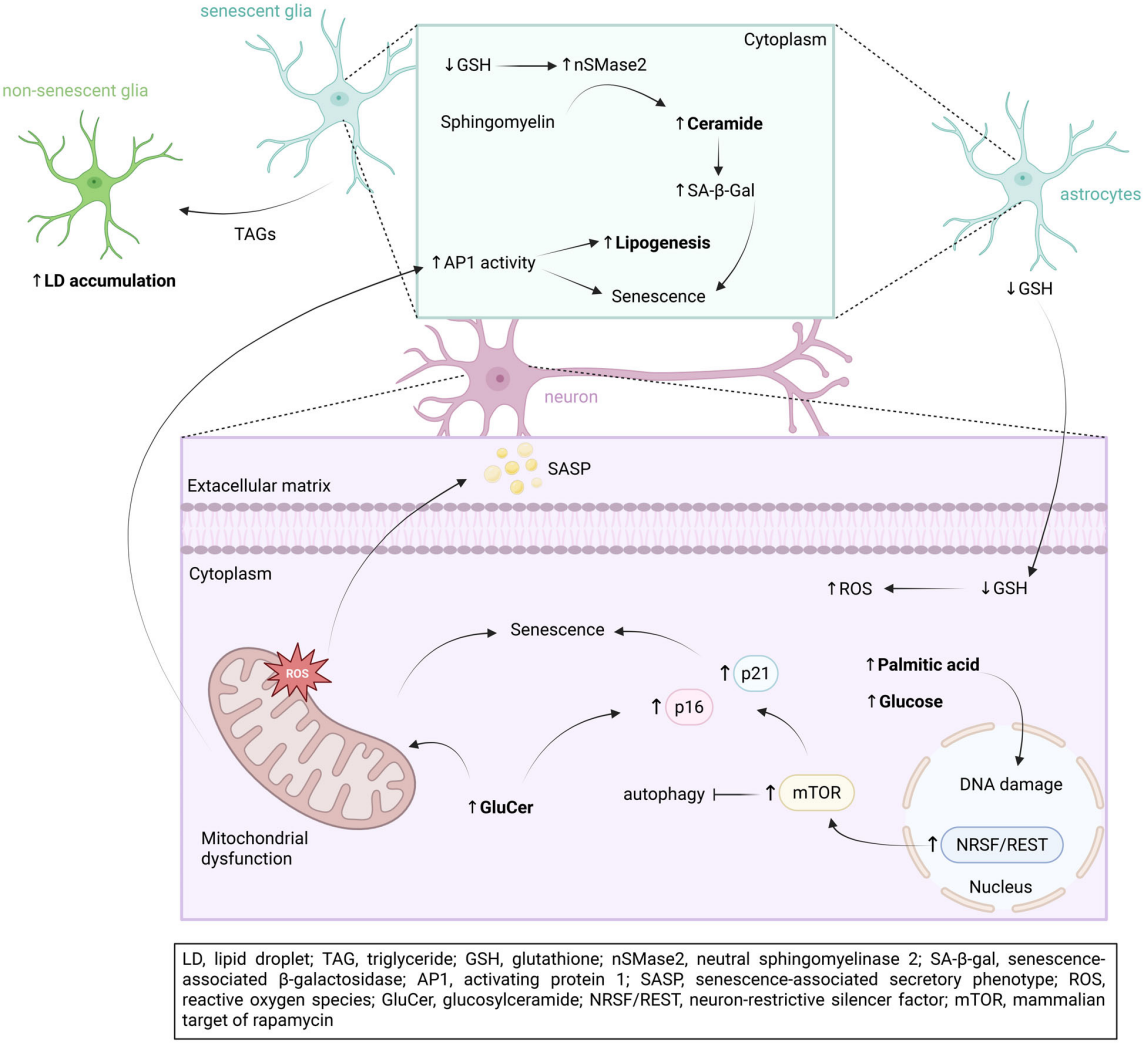
Dysregulated lipid metabolism in neuronal senescence. High glucose and palmitic acid increase DNA damage and induce senescence, marked by increased p16/p21 levels, in PC12 cells and primary mouse cortical neurons, through upregulation of neuron‐restrictive silencer factor (NRSF/REST), which in turn activates mTOR signalling and suppresses autophagy. In dopaminergic neurons, GluCer accumulation leads to elevated reactive oxygen species (ROS) production, increased p16/p21 levels and SASP release, driving senescence. Neuronal mitochondrial dysfunction increases AP1 activity in senescent glia, marked by increased lipogenesis that promotes lipid droplets (LD) accumulation in nonsenescent glia. In astrocytes, glutathione (GSH) precursors are provided to neurons. Oxidative stress reduces GSH levels, preventing the inhibition of nSMase2 and leading to its activation and subsequent ceramide elevation, increased SA‐β‐Gal activity and senescence markers. Meanwhile, reduced GSH from astrocytes impairs the ability of neurons to neutralise ROS. (Artwork created with BioRender.com).

Alterations in lipid metabolism are consistently observed as a consequence of glial senescence. A recent study in *Drosophila* demonstrated that neuronal mitochondrial dysfunction triggers senescence and LD accumulation in glial cells, aligning with previous findings in mammalian systems [42, 43]. With ageing, activating protein 1 (AP1)^+^ glia begin to exhibit classical hallmarks of senescence, including increased SASP, elevated SA‐β‐Gal activity, cell cycle arrest and upregulation of senescence‐related genes. Notably, while senescent glial cells show enhanced lipogenesis, LD accumulation is more pronounced in nonsenescent glia, likely due to their active metabolism and transfer of triglycerides (TAGs) to neighbouring senescent cells, which may serve as a protective mechanism against ROS (Fig. 1) [42]. In accordance, disruption of LD formation, via manipulation of lipogenesis or lipolysis, was found to have detrimental effects, suggesting a protective function for LDs during glial senescence, sequestering lipids from peroxidation [42, 44]. However, further research is required to clarify the mechanisms underlying differential LD accumulation between AP1^+^ and AP1^−^ glia and its functional significance [43]. Conversely, in obese mouse models, lipid accumulation is observed specifically in senescent glial cells [45]. Here, it is not the absolute lipid levels, but the proportion of lipid‐laden senescent cells that impairs neurogenesis [45]. These studies underscore the context‐dependent roles of LDs in glial senescence, pointing to a complex interplay between lipid storage, glial senescence and neuronal health.

On the contrary, lipid‐induced senescence has been documented in other brain cells. In astrocytes, oxidative stress reduces glutathione (GSH) levels, preventing the inhibition of the enzyme neutral sphingomyelinase 2 (nSMase2) that converts sphingomyelin to ceramide. This in turn leads to its subsequent activation and accumulation of ceramide, resulting in induction of senescence, as marked by an increase in SA‐β‐Gal activity and senescence markers [46]. GSH precursors are provided from astrocytes to neurons; thus, reduced GSH from astrocytes impairs the ability of neurons to neutralise ROS, contributing to neuronal dysfunction (Fig. 1) [46]. Moreover, a diabetic‐like environment, simulated by high glucose and palmitic acid, induces senescence in PC12 cells and primary mouse cortical neurons, as evidenced by increased DNA damage, SA‐β‐Gal activity, and elevated levels of p16 and p21 [47]. This effect was mediated by the upregulation of neuron‐restrictive silencer factor (NRSF/REST), which activated mammalian target of rapamycin (mTOR) signalling and suppressed autophagy (Fig. 1) [47]. Furthermore, accumulation of glucosylceramide (GluCer) has been shown to induce senescence specifically in dopaminergic neurons, marked by an elevation in SA‐β‐Gal activity, p21 and p16 levels, and SASP release [48], suggesting a mechanistic link between lipid dysregulation and neuronal senescence in driving the progressive loss of dopaminergic neurons (Fig. 1) [49]. However, in most studies, there are no established complete causal chains from lipid dysregulation to senescence induction, as research is mostly focused on endpoint measurements of senescence markers—p21 and p16—rather than mechanistic pathways.

Current studies increasingly focus on elucidating the mechanisms by which LDs drive neuronal senescence. Accumulation of low‐density lipoprotein cholesterol (LDL‐C) induces lipotoxicity, DNA damage and senescence in recruited neural stem cells, preventing repair of the tumour‐induced nerve damage in the hypothalamic third ventricle floor [50]. This lipotoxicity is mediated by LD accumulation and activation of integrated stress response (ISR) signalling, particularly through the p21 signalling pathway, which inhibits CDK complexes, enforcing a cell cycle checkpoint by causing G1 arrest [50]. Similarly, arsenic exposure has been shown to induce dose‐dependent senescence in both neurons and glial cells, particularly in brain regions critical for social memory [51]. These senescent cells are marked by LD accumulation, with astrocytes and microglia showing greater LD content than neurons [51]. This disparity may be attributed to increased ROS production and lipid peroxidation in neurons, leading to transport of peroxidised lipids to other cells. Finally, it has been shown that radio‐ and chemotherapy activate asparagine endopeptidase (AEP) in tumour cells, increasing prostaglandin E_2_ synthesis, which acts on nearby neurons, in turn inducing the expression of senescence‐associated genes, such as Bmp5, Pax3 and Foxl2, which lead to the activation of canonical p38/ERK and p53/p21 signalling cascades. As a result, neurons undergo senescence, characterised by reduced excitability, apoptosis resistance and secretion of SASP factors, which in turn reinforce tumour survival and therapy resistance [52].

## Lipid metabolism and senescence in the ageing brain

The composition and abundance of lipids in the brain undergo significant changes during ageing. Lipidomic analyses of whole brains from aged mice have revealed increased levels of phospholipids and sphingomyelin, compared with younger mice [53]. Region‐specific studies across 13 distinct brain areas in both young and older individuals have further highlighted marked differences in fatty acid (FA) profiles, particularly in the inferior temporal cortex and the cingulate gyrus [54]. These alterations primarily involve monounsaturated and polyunsaturated FAs (MUFAs and PUFAs), consistent with region‐specific PUFA shifts in aged senescence‐accelerated prone mice [54, 55]. Notably, elevated omega‐3 FA levels are associated with improved memory performance, emphasising the functional relevance of these lipid changes [56]. In parallel, ageing has also been linked to alterations in lipid rafts, membrane microdomains essential for neuronal signalling [57]. Together, these findings underscore the complexity and regional specificity of lipid remodelling in the ageing brain and its potential role in age‐related cognitive decline.

In neurons, age‐related changes in sphingolipid and cholesterol metabolism significantly affect cellular function and survival. For instance, genetic inhibition of serine palmitoyltransferase (SPT), a key enzyme in *de novo* ceramide biosynthesis, reverses mitochondrial dysfunction and Ca^2+^ dyshomeostasis in aged cortical neurons [58], highlighting the contribution of ceramides to neuronal senescence. In parallel, increased sphingomyelin levels in aged hippocampal neurons have been shown to support the stability of the tropomyosin receptor kinase B (TrkB), a key component in neurotrophic signalling [59]. Additionally, age‐related upregulation of cholesterol‐24‐hydroxylase, a catabolic enzyme, leads to reduced membrane cholesterol, which paradoxically enhances TrkB signalling, despite declining levels of brain‐derived neurotrophic factor (BDNF) [60, 61]. Given that BDNF/TrkB signalling is a pro‐survival pathway exploited by senescent neurons to maintain viability [62], these lipid‐mediated regulatory mechanisms are particularly significant. However, excessive ceramide accumulation or cholesterol loss can also impair insulin signalling, contributing to insulin resistance and cognitive decline during ageing [61, 63]. Collectively, these findings highlight the regulatory role of lipids in sustaining neuronal survival in the ageing brain.

Glial cells in both the CNS and the peripheral nervous system (PNS), primarily Schwann cells, undergo substantial changes in lipid metabolism with ageing, affecting neuronal survival and communication. The lipolysis‐stimulated receptor (LSR), expressed in both neurons and glia, plays a critical role in maintaining cholesterol homeostasis and glia–neuron cross talk [64]. In aged mouse brains, LSR expression exhibits region‐specific changes, suggesting that cholesterol may assume distinct functional roles with ageing, depending on brain region and cell type [64]. In *Drosophila*, knockdown of the glial uridine 5′‐diphospho‐glucuronosyltransferase (UDP)‐glycosyltransferase 35 (Ugt35b) leads to a reduction in key lipids of glycerolipid and glycerophospholipid metabolism, such as phosphatidylethanolamine (PE) and phosphatidylcholine (PC) [65]. Ultimately, these lipid changes disrupt LD formation, leading to accelerated senescence [65]. In the PNS, impaired lipid metabolism in Schwann cells is associated with age‐related dysfunction. Specifically, downregulation of cholesterol and FA metabolism in these cells is either the cause or result of demyelination, observed in both normal ageing and age‐related neuropathies [66]. Additionally, recent findings report an increase in lipofuscin, known as ‘age‐pigment’ but also a cellular senescence marker [67], p21 and p16 levels and the expression of IL6 cytokine in human dorsal root ganglia neurons with ageing [68]. While all these studies underscore a strong correlation between lipid alterations and senescence during ageing, further research is needed to resolve whether lipid alterations are the trigger of senescence or the by‐product of the senescent phenotype.

## Lipid metabolism in senescence‐associated neurodegenerative diseases

**Fig. 2:**
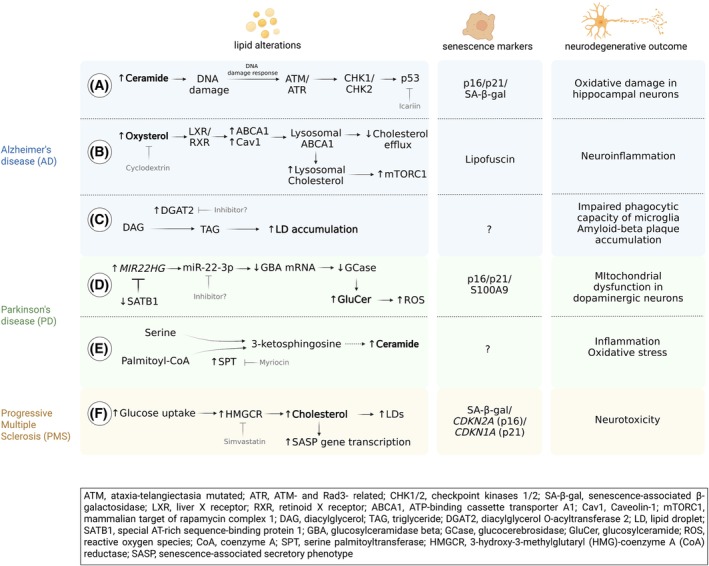
Lipid alterations and therapeutic interventions in senescence‐associated neurodegenerative diseases. (A) In Alzheimer's disease (AD), elevated ceramides activate a DNA damage response that likely engages the ATM/ATR‐CHK1/2‐p53 pathway, leading to increased expression of senescence markers (p16, p21 and SA‐β‐gal) and oxidative damage in hippocampal neurons. Modulating p53 signalling with icariin mitigates lipid‐induced senescence. (B) Accumulation of oxysterols activates the nuclear receptors, liver X receptor (LXR) and retinoid X receptor (RXR), upregulating ABCA1 and Cav1, resulting in persistent translocation of ABCA1 in lysosomes, impaired cholesterol efflux, triggering cholesterol and lipofuscin accumulation, mTORC1 activation, and ultimately neuroinflammation. Cyclodextrin treatment reduces oxysterol, prevents ABCA1 lysosomal sequestration and attenuates senescence. (C) Increased DGAT2 activity promotes lipid droplets (LD) accumulation in microglia, which impairs phagocytosis and enhances accumulation of amyloid‐beta plaques. No senescence markers have been reported, while DGAT2 inhibition may reduce LD accumulation and mitigate neurodegenerative effects. (D) In Parkinson's disease (PD), age‐related downregulation of SATB1 increases miR‐22‐3p from *MIR22HG*, suppressing GBA expression and GCase activity. This causes GluCer accumulation, mitochondrial dysfunction, elevated reactive oxygen species (ROS) and upregulation of senescence markers (p16, p21 and S100A9). Inhibiting miR‐22‐3p may alleviate these effects. (E) Increased serine palmitoyltransferase (SPT) activity elevates ceramide levels, promoting inflammation and oxidative stress; SPT inhibition by myriocin prevents ceramide accumulation. No senescence markers have been reported. (F) In progressive multiple sclerosis (PMS), senescence‐induced stem cells show elevated glucose uptake and3‐hydroxy‐3‐methylglutaryl (HMG)‐coenzyme A (CoA) reductase (HMGCR) activity, driving cholesterol‐dependent SASP activation and increased SA‐β‐gal, leading to neuronal toxicity. Simvastatin modulates this pathway, shifting the SASP towards a cytoprotective profile. (Artwork created with BioRender.com).

Disruption in lipid metabolism is a well‐established hallmark of neurodegenerative disorders [29, 69, 70]. For instance, cholesterol levels increase in the brains of both Alzheimer's disease (AD) patients and mouse models [71], and notably, these changes are directly correlated with AD severity [72]. Additionally, changes in the levels of PCs, TAGs and sphingolipids are involved in Parkinson's disease (PD) progression and dyskinesia severity [73, 74]. Here, we highlight key alterations in lipid homeostasis, encompassing lipid synthesis, transport and storage, that are implicated in neural senescence and neurodegeneration (Fig. 2).

## Lipid synthesis

Alterations in lipid synthesis are recognised as important contributors to cellular senescence and progression of neurodegenerative disorders. Induced neural stem cell (iNSCs) lines, derived from fibroblasts of patients with progressive multiple sclerosis (PMS), are senescent, as shown by upregulated *CDKN2A* (p16) and *CDKN1A* (p21) levels. Interestingly, these cells are also hypermetabolic, characterised by increased glucose uptake, leading to increased cholesterol synthesis and LD accumulation, inducing the neurotoxic release of SASP (Fig. 2) [75]. Similarly, single‐cell RNA sequencing of postmortem brains from AD patients revealed that microglia display dysregulated cholesterol homeostasis, including synthesis, metabolism and storage, linked to a senescence phenotype [76]. Beyond just associations, ceramide alterations can trigger senescence‐related neurodegeneration. Elevated ceramide levels induce senescence by triggering a DNA damage response that activates the p53 pathway [77]. It is likely this occurs through stimulation of the upstream kinases ATM and ATM‐ and Rad3‐related (ATR), and their downstream effectors Checkpoint kinases 1 and 2 (CHK1/2) [78], leading to AD‐related changes in hippocampal neurons, such as oxidative damage (Fig. 2) [77]. Notably, increased ceramide levels have also been reported in postmortem brain tissues from AD patients, although it remains unclear whether these lipid alterations occur due to altered gene expression in the brain or represent compensatory responses [79]. Conversely, reduced ceramide synthesis, due to ceramide synthase 1 (CerS1) deficiency, leads to accumulation of the senescence marker lipofuscin and degeneration of Purkinje neurons, with abnormal dendritic development in these neurons, highlighting the importance of sphingolipids in dendritic growth [80]. These findings suggest that imbalances in cholesterol and ceramide levels are associated with neuronal and glial senescence, ultimately contributing to neurodegeneration, but mechanistic insights on how these lipidomic alterations arise during senescence are missing.

## Lipid transport

Lipid transport in the CNS is mainly mediated by ATP‐binding cassette (ABC) transporters, namely subfamily A member 1 (ABCA1) and G1 (ABCG1), and Apolipoprotein E (APOE). Recent studies have demonstrated that neurons, astrocytes and microglia from postmortem human AD brains carrying the APOE4 allele exhibit elevated markers of senescence, along with increased accumulation of oxysterols [81]. This accumulation leads to increased expression of ABCA1 and Caveolin‐1 (Cav1), resulting in persistent translocation of ABCA1 to lysosomes and disruption of cholesterol transport. Subsequently, cholesterol, as well as the senescence marker lipofuscin, is accumulated in lysosomes, leading to mTOR complex 1 (mTORC1) activation and contributing to cellular senescence and neuroinflammation (Fig. 2) [81]. Consistent with this, disrupted cholesterol transport in oligodendrocytes caused by APOE4 impairs myelination and contributes to cognitive dysfunction, supporting a causal relationship between dysregulation in cholesterol metabolism and the severity of AD progression [82]. The role of cholesterol transport in the induction of senescence has been further underscored by a study in *Abca1/g1*‐knockout bone‐marrow‐derived macrophages (BMDMs), where impaired cholesterol efflux results in intracellular cholesterol accumulation [83]. This activates liver X receptor (LXR), upregulating both ABCA1/G1 and CD38, an NAD^+^ hydrolase, depleting NAD^+^ levels in macrophages, triggering senescence and promoting features associated with age‐related macular degeneration (AMD) [83]. Similarly, knockout of ABCA1/G1 in retinal rod photoreceptors leads to cholesterol efflux deficiency and thus cholesterol accumulation, which induces cellular senescence, as shown by elevated p16/p21 markers and increase of SASP factors like TNF‐α, contributing to retinal neurodegeneration [84]. Collectively, excessive cholesterol accumulation due to impaired transport may be a major inducer of senescence and a key contributor to neurodegenerative disease pathogenesis.

## Lipid storage

Senescence‐associated neurodegenerative phenotypes are also characterised by increased lipid storage and accumulation. Notably, glucosylceramidase beta 1 (GBA), which encodes for the lysosomal enzyme glucocerebrosidase (GCase), is a genetic risk factor for PD [49, 85]. Reduction of GCase is caused by an age‐related decrease in the transcriptional regulator Special AT‐rich sequence‐binding protein 1 (SATB1), which normally represses *MIR22HG* expression. The micro‐RNA miR‐22‐3p targets GBA mRNA, reducing its transcription and subsequently GCase activity. These events lead to the accumulation of glycolipids, such as GluCer and glucosylsphingosine (GluSph), which contribute to early pathogenic events of PD [85]. This is in accordance with findings supporting that GluCer accumulation leads to increased ROS in mitochondria and dopaminergic neuronal senescence, marked by increased p16/p21 and S100A9 (Fig. 2) [48, 49]. In addition, postmortem analyses of PD brains have shown that intracellular neutral lipid accumulation is prominent in dopaminergic neurons and midbrain microglia, but not in neighbouring astrocytes, indicating a cell type‐specific dysregulation of lipid homeostasis within the substantia nigra [86]. Similarly, in AD, microglia exposed to amyloid‐beta form LDs driven by an upregulated activity of diacylglycerol O‐acyltransferase 2 (DGAT2), which catalyses the final step in TAG synthesis by converting DAG and acyl‐CoA into TAGs [87]. The resulting lipid‐loaded microglia display impaired phagocytic capacity, thereby exacerbating amyloid‐beta plaque accumulation. Notably, DGAT2 inhibition reduces LD formation and restores microglial phagocytosis, implicating lipid metabolism in microglial dysfunction and AD pathology (Fig. 2) [87]. Although alterations in LD accumulation have been associated with cellular senescence, this study did not correlate these phenotypes with senescence. Additionally, microglia from postmortem AD brain tissue with the APOE4/4 genotype are characterised by upregulated activity of acyl‐CoA long‐chain family member 1 (ACSL1), an enzyme involved in LD biogenesis, resulting in increased LD accumulation [88]. Importantly, conditioned media from LD accumulating microglia can induce tau phosphorylation and neurotoxicity in an APOE‐dependent manner, suggesting that these lipid‐laden microglia secrete neurotoxic factors that contribute to neurodegeneration [88]. Tau‐containing neurofibrillary tangles have already been reported to drive neuronal senescence, marked by elevated p16 and p21 and mitochondrial dysfunction, in AD mouse models [89]. Complementary work has shown the reverse relationship; senescent astrocytes and microglia can promote tau pathology and neurodegeneration through extracellular signalling via the SASP, rather than by altering tau production [90]. Thus, there is a bidirectional feed‐forward loop between senescence in the brain and tau pathology in neurodegeneration. Collectively, these findings underscore the critical role of lipid accumulation across various brain cell types in driving cellular senescence and functional decline in neurodegenerative diseases.

## Lipid metabolism as a therapeutic target in senescence‐related neurodegenerative disorders

Studies in AD and PD mouse models underscore a causal role of senescence in the progression of neurodegenerative disorders. Thus, the use of senolytics for improving the behaviour of such transgenic animals has been widely investigated. For instance, senolytic treatment with dasatinib and quercetin selectively clears senescent oligodendrocyte precursor cells in an AD mouse model, reducing neuroinflammation, Aβ plaques and cognitive decline [91]. However, there are still many challenges in the use of senolytics, such as the lack of specificity and side effects [9, 92]. Given these limitations, targeting metabolic pathways that drive neuronal senescence, particularly lipid metabolism, might offer novel therapeutic opportunities for age‐related neurodegenerative disorders (Fig. 2).

Statins are widely used medications that lower low‐density lipoprotein (LDL). Simvastatin, an FDA‐approved inhibitor of 3‐hydroxy‐3‐methylglutaryl (HMG)‐coenzyme A (CoA) reductase (HMGCR), has shown efficacy in reducing neurotoxicity in senescent neural stem cells from PMS patients by altering the cholesterol‐induced SASP profile (Fig. 2) [75]. In clinical trials, a high dose of simvastatin had previously shown to attenuate brain atrophy in secondary PMS, though the mechanism of action in this case was independent of peripheral cholesterol lowering [93]. Moreover, cyclodextrin compounds, particularly hydroxypropyl‐β‐cyclodextrin (HPCD), have emerged as promising agents for restoring cholesterol efflux and accumulation. In APOE4 models, cyclodextrin treatment reduced oxysterol levels, prevented ABCA1 lysosomal sequestration and attenuated senescence markers (Fig. 2) [81]. Nevertheless, beyond pharmacological interventions targeting cholesterol regulation, dietary or lifestyle approaches may also contribute to restoring cholesterol homeostasis and promoting myelin maintenance, thereby enhancing cognitive reserve in APOE4 carriers [82]. Another drug, myriocin, which blocks serine palmitoyltransferase (SPT) that is involved in *de novo* ceramide biosynthesis by catalysing the conversion of palmitoyl‐CoA and serine to 3‐ketosphingosine, has shown beneficial effects in a cellular PD model through autophagy activation and reduction of lipid peroxidation (Fig. 2) [94]. The major limitation of this study is that the experimental system represents acute stress, while in PD patients, the cellular response could be different, as α‐synuclein is gradually accumulated. Similarly, targeting other sphingolipid pathways, including nSMase2 inhibition, reduces ceramide‐induced senescence in astrocytes [46]. Finally, icariin, a natural flavonoid glucoside, is a promising therapeutic agent for AD, acting against ceramide‐induced neuronal senescence by inhibiting the p53 pathway [77]. Specifically, icariin suppresses downstream p21‐mediated cell cycle arrest, leading to reduced ROS production and improved mitochondrial and synaptic function, thereby attenuating age‐related phenotypes in cortical neurons (Fig. 2) [77].

While these interventions show promise, the essential roles of lipids in proper brain function necessitate cautious evaluation of potential risks. For instance, simvastatin can impair synaptic plasticity in the hippocampus and cause memory deficits through cholesterol reduction [95], as cholesterol is essential for synapse formation and myelination. This is in accordance with clinical trials showing a negative association between the use of statins and cognitive function [96, 97], which warrants further exploration. In addition, targeting ceramide metabolism in the brain might exacerbate disease phenotypes. Notably, treatment with glucosylceramide synthase inhibitors can lead to ceramide accumulation, which can be toxic in a cell‐dependent manner, with astrocytes being less affected than neurons and microglia [98]. Therefore, novel therapeutic approaches targeting lipid metabolism in senescence‐related neurodegeneration present both an opportunity but also a challenge. Finally, it is important to note that it is not only pharmacological and genetic interventions that have been shown to delay cellular senescence and reduce the burden of age‐related disorders, but also lifestyle modifications, such as physical exercise and caloric restriction.

## Concluding remarks

Here, we survey the emerging landscape of lipid metabolism in neuronal senescence, highlighting the complex interplay between metabolic dysregulation, cellular senescence and neuronal health. We demonstrate that alterations in lipid homeostasis represent both drivers and consequences of the senescent state in brain cells, with cell type‐specific patterns reflecting the metabolic specialisation of neurons and glia. Key unanswered questions in the field remain: What are the upstream regulators of lipid‐mediated neuronal senescence? Can modulating lipid metabolism selectively target senescent cells without harming neuronal function? How do we distinguish pathological from protective lipid accumulation? Understanding the role of lipid metabolism in neuronal senescence holds promise for yielding transformative insights into promoting healthy brain ageing and treating age‐related neurological disorders.

## Conflict of interest

The authors declare no competing interests.

## Author contributions

DT and NT conceptualised the work. DT and EP wrote the draft of the manuscript. EP prepared all figures. NT revised and edited the manuscript.
